# Motion and dural sac compression in the upper cervical spine during the application of a cervical collar in case of unstable craniocervical junction—A study in two new cadaveric trauma models

**DOI:** 10.1371/journal.pone.0195215

**Published:** 2018-04-06

**Authors:** Shiyao Liao, Niko R. E. Schneider, Petra Hüttlin, Paul A. Grützner, Frank Weilbacher, Stefan Matschke, Erik Popp, Michael Kreinest

**Affiliations:** 1 BG Trauma Center Ludwigshafen, Department of Trauma Surgery and Orthopaedics, Ludwigshafen, Germany; 2 University Hospital Heidelberg, Department of Anesthesiology, Heidelberg, Germany; Medical College of Wisconsin, UNITED STATES

## Abstract

**Background:**

Unstable conditions of the craniocervical junction such as atlanto-occipital dislocation (AOD) or atlanto-axial instability (AAI) are severe injuries with a high risk of tetraplegia or death. Immobilization by a cervical collar to protect the patient from secondary damage is a standard procedure in trauma patients. If the application of a cervical collar to a patient with an unstable craniocervical condition may cause segmental motion and secondary injury to the spinal cord is unknown.

The aim of the current study is (i) to analyze compression on the dural sac and (ii) to determine relative motion of the cervical spine during the procedure of applying a cervical collar in case of ligamentous unstable craniocervical junction.

**Methods and findings:**

Ligamentous AOD as well as AOD combined with ligamentous AAI was simulated in two newly developed cadaveric trauma models. Compression of the dural sac and segmental angulation in the upper cervical spine were measured on video fluoroscopy after myelography during the application of a cervical collar. Furthermore, overall three-dimensional motion of the cervical spine was measured by a motion tracking system.

In six cadavers each, the two new trauma models on AOD and AOD combined with AAI could be implemented. Mean dural sac compression was significantly increased to -1.1 mm (-1.3 to -0.7 mm) in case of AOD and -1.2 mm (-1.6 to -0.6 mm) in the combined model of AOD and AAI. Furthermore, there is a significant increased angulation at the C0/C1 level in the AOD model. Immense three-dimensional movement up to 22.9° of cervical spine flexion was documented during the procedure.

**Conclusion:**

The current study pointed out that applying a cervical collar in general will cause immense three-dimensional movement. In case of unstable craniocervical junction, this leads to a dural sac compression and thus to possible damage to the spinal cord.

## Introduction

The craniocervical junction (CCJ) consists of occiput, atlas, axis, and a complex system of supporting ligaments and synovial joints [1, 2]. CCJ injuries have been identified in 30% of 300 patients with cervical spine injuries [3], and CCJ injuries were postmortem diagnosed in 22.6% of 312 deceased traffic victims [4]. Among these, atlanto-occipital dislocation (AOD) is considered as a severe and fatal injury in cervical spine that only few patients survive [5–11]. The incidence of AOD may be as high as 6% up to 10% in fatal cervical spine injuries [12, 13].

The purely severe ligamentous injury of CCJ may occur as an isolated AOD, or as a combined injury. An increasing number of patients suffering from AOD combined with atlanto-axial instability (AAI) have been reported [9, 10, 14–17]. The combined distractive injury may result from a high-energy trauma [10]. A previous study suggested that the combination of AOD and AAI was diagnosed in 35% of patients with CCJ distractive injuries [17]. Distractive CCJ injury is frequently associated with significant neurological deficits and a high rate of mortality as a consequence of spinal cord injury, brainstem injury or vascular injury [5, 16, 18]. Since improvements of cervical spine immobilization, increased recognition and more progressive treatment, mortality rate and neurological deficit of distractive CCJ injury have diminished in recent years [7, 19].

Traditionally, cervical spine immobilization is a standard procedure in trauma patients and is recommended by relevant treatment protocols [20] and guidelines [21]. As a common immobilization technique, cervical collars have been widely applied in pre-hospital care and emergency department care, specifically intended to restrict the cervical spine in a neutral position and to protect the cervical spinal cord from secondary injury.

However, there is no supporting evidence that cervical collars can effectively protect the spine from intervertebral motion within unstable CCJ [22]. Furthermore, a study performed by Lador et al. demonstrated that the application of a collar may generate intervertebral motions and may shift the axis of rotation of the unstable cervical spine [23]. Another cadaveric study shows an increased distraction after the application of a cervical collar in the presence of severe distractive CCJ injury [24]. Moreover, the application of a cervical collar is associated with further disadvantages such as impeded airway management [25, 26] and compression of the jugular veins [27] leading to a significant increase in intracranial pressure [28–32]. Thus, recent immobilization protocols are more restrictive towards the use of cervical collars [33, 34]. Current literature also reports that there are some deficits concerning the practical skills of a collar’s application [35] that may lead to reduced spinal immobilization [36]. If the procedure of applying a cervical collar to a patient with unstable CCJ may cause segmental motion and secondary injury to the spinal cord is unknown.

Thus, the aim of the current study is (i) to analyze compression on the dural sac and (ii) to determine relative motion of the cervical spine during the procedure of applying a cervical collar in case of unstable CCJ in two new cadaveric models.

## Materials and methods

The study proposal was approved by Ethics Committee of the State Medical Association Rhineland-Palatinate (Mainz, Germany; Registry No. 837.156.16). The study was registered in the German Clinical Trials Register (ID: DRKS00010499; S1 Trial Register).

We recruited fresh cadavers from the body donation program at Heidelberg University. Members of the public donated their body after death and provided written informed consent for the cadavers to be used in medical research and education.

### Eligibility criteria

Fresh cadavers were frozen shortly after mortem and thawed to room temperature for simulating the elasticity of joints and soft tissues in living situation. Recent studies of cadaver biomechanics postulate no significant differences between fresh cadavers and patients towards cervical spine motion [37–40]. Cadavers with a postmortem interval of less than 5 days were eligible for the study. Before enrolled, we checked exclusion criteria (cervical spine disease, cervical spine surgical history, neck trauma) by reviewing the medical records of the donors. Furthermore, cervical spine fluoroscopy was performed on every cadaver to exclude degenerative diseases or injuries to the cervical spine.

### Cadaveric CCJ instability models

To our knowledge, there are only few cadaver studies that have formally reported to create an AOD model or a combined model of AOD and AAI in an intact cadaver so far [23]. Our study developed two cadaveric models by referring to the anatomical studies about the CCJ.

#### AOD cadaveric model

**Fig 1:**
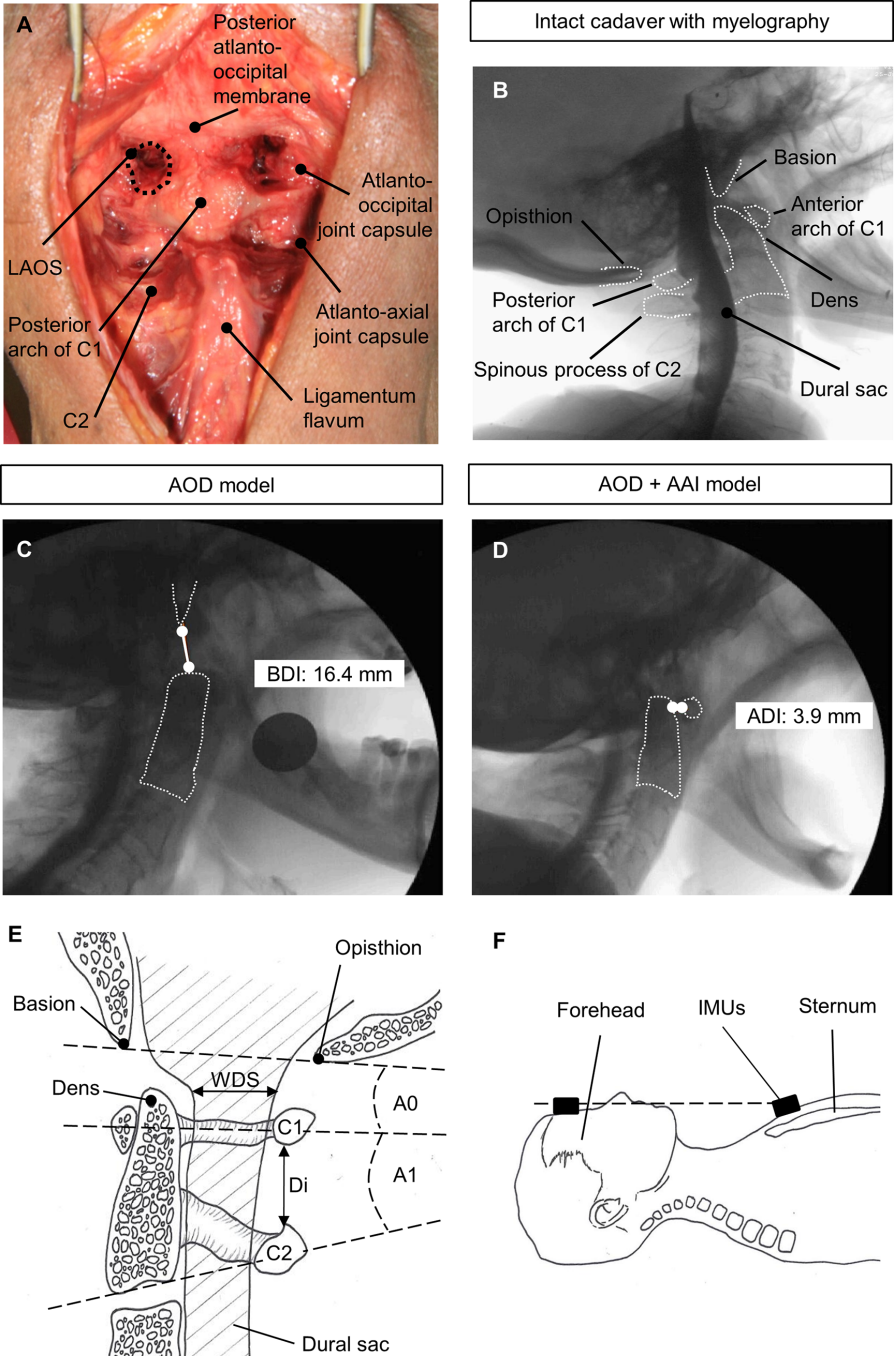
The cadaveric model: Creation, confirmation and measurement. View of the anatomic landmarks to create the cadaver models (A). Video fluoroscopy and myelography allows analyzation of all bony structures and the dural sac (B) as well as the confirmation of AOD model (C) and AOD + AAI model (D). Schematic drawing about the measurements in the upper cervical spine (E) and the placement of the wireless human motion tracker system (F).

Atlanto-occipital (C0/C1) articulation stability is maintained by the most upper crucial ligaments involving the alar ligaments, the tectorial membrane, and the atlanto-occipital joint capsule [41, 42]. Disruption of these structures is required in AOD. A posterior surgery was performed on upper cervical spine (Fig 1A). Cadaver was positioned prone in hyperflexion. A midline incision of approximately 12 cm was made starting at the occiput (Fig 1A). To expose occiput and anterior arch of C1, atlanto-occipital membrane was severed and removed (Fig 1A). Then, atlanto-occipital joint capsules have been prepared (Fig 1A); the joints have been opened and distracted by a small chisel (not shown in Fig 1A). Afterwards, the dural sac was protected to the medial side and tectorial membrane was cut horizontally at the level between basion and dens (Fig 1E) by a small scalpel via both lateral atlanto-occipital spaces (LAOS, Fig 1A).

The instability of the model was confirmed by lateral video fluoroscopy during flexion and extension. According to the consensus statement of measurement for upper cervical spine injuries [43–45], a basion-dental interval (BDI) of more than 12 mm was diagnosed as AOD (Fig 1C). BDI is measured through the distance between basion and tip of dens [43–45].

#### Combined model of AOD and AAI

Transverse ligament serves as a stabilizer of atlanto-axial (C1/C2) articulation by attaching the odontoid process to the anterior arch of C1 [46–48]. The complete rupture of transverse ligament causes an AAI. A further posterior surgery was performed on the cadavers with AOD. Atlanto-occipital joints were distracted again. Then, the dural sac was protected to the medial side and transverse ligaments were cut vertically at the level of the dens by a small scalpel via both lateral atlanto-occipital spaces (LAOS, Fig 1A). Additionally, atlanto-axial joint capsules were opened and distracted by a small chisel (not shown in Fig 1A).

The combined model of AOD and AAI was confirmed by lateral video fluoroscopy during flexion and extension. If atlanto-dens interval (ADI) is measured more than 3 mm AAI is diagnosed (Fig 1D) as firstly described by Hinck et al. [49] and widely recommended [43, 50–52]. The distance at the midpoint line between the posterior border of anterior arch of C1 and anterior border of the dens was measured as ADI.

### Myelography and video fluoroscopy

The cadavers were positioned prone and a mini-incision surgery was performed to expose dural sac in upper thoracic spine via posterior approach. A subarachnoid space puncture was performed and a tube was placed cranially. Contrast medium (Optiray, 300 mg/ml, Mallinckrodt, Germany) was pump injected through the tube into dural sac. Myelography under video fluoroscopy (Veradius C-Arm, Philips, Netherlands) was used (Fig 1B) to directly measure the real-time changes of the dural sac’s width during the procedure of applying a cervical collar. Thus, myelography provides clear information about dural sac compression caused by soft tissues or bony structures [53, 54].

During fluoroscopy, distance of the C-Arm to the cadavers’ cervical spine was set to 30 cm. Central ray orientation was standardized and a measuring reference was fixed at every cadaver.

### Intervention

All cervical collars (Stifneck Select, Laerdal Medical, Puchheim, Germany) were adjusted to their correct size before the procedure of application started. All collar applications were performed in random order by two emergency physicians who attended a special training on the treatment of trauma patients [55]. The intervention providers were blinded from fluoroscopic images and wireless human motion tracker system during the entire experiment. The baseline data of all CCJ conditions had been gathered prior to application of the intervention.

### Measurements

#### Width of dural sac

This study was primarily designed to measure the change of the dural sac’s width (WDS). We assessed the dural sac space by directly measuring width of dural sac in sagittal plane on myelographic views. WDS was defined as the narrowest distance of the dural sac in the injured level (C0/C1 or C1/C2) during the procedure of applying a cervical collar (Fig 1E). Negative values of change in WDS represent the amount of dural sac compression.

#### Angulation

The angle of intersection of reference lines on each vertebral body was measured as angulation of each cervical spine segment [37, 56, 57]. Angulation at C0/C1 segment (Fig 1E, A0) was measured through the angle of intersection between the line drawn from basion to opisthion and midpoint line of C1. Angulation at C1/C2 segment (Fig 1E; A1) was measured through the angle of intersection between midpoint line of C1 and inferior endplate line of C2. The neutral position before any manipulation was recorded as baseline, we defined flexion as positive values and extension as negative values.

#### Distraction

Distraction at C1/C2 level was measured through the perpendicular distance between the posterior ring of C1 and the superior spinolaminar line of C2 [56] (Fig 1E; Di). The neutral position before any manipulation was recorded as baseline.

#### Overall motion of cervical spine

Overall motion of cervical spine during the procedure of cervical collar application was assessed by measuring the changes of the head relative to the trunk [58] by wireless human motion tracker system (Xsens Technologies, Enschede, Netherlands). The 3-Dimension measurements involve extension/flexion as well as rotation and lateral bending. Two inertial measurement units (IMUs) were placed on the forehead and sternum of each cadaver, respectively (Fig 1F). The neutral state of each cadaver positioned supine on a table before maneuvers was marked as baseline. Flexion was defined as positive values. We assessed rotation and lateral bending using absolute values, regardless of right or left rotation and right or left lateral bending.

### Sample size and statistics

#### Sample size calculation

The data from preliminary pilot experiments (data not shown) demonstrate standard deviation (SD) value of 0.2 mm for the change of dural sac’s width during the procedure of applying a cervical collar. Mid-sagittal diameter of subarachnoid space at C0/C1 level is 8–9 mm [59]. A sample size of six cadavers for each model was calculated to detect a 0.3 mm difference for the change in the width of the dural sac during the procedure of applying a cervical collar (α = 0.05, power of 90%).

#### Statistical analysis

Wilcoxon signed rank test was used to make pairwise comparisons in stable versus unstable CCJ conditions. Mann-Whitney test was used to make non-paired comparisons between C0/C1 segment and C1/C2 segment. α < 0.05 was set as significant. All values are reported as median (range). Data were analyzed using SPSS Statistics 22.0 (IBM, Ehningen, Germany).

## Results

### Cadaveric CCJ instability models

In a pilot study, surgical technique was first tested and improved on two formalin-fixed cadavers (data not included in this study). In the current study, six fresh cadavers (one female and five male) were involved. Age at death was 82 years (76–100 years). All cadavers showed a physiological range of mobility of the cervical spine. After measuring intact cadavers (stable CCJ condition), the ligamentous AOD model could be implemented successfully in all six cadavers confirmed by BDI (Fig 1C). Following the measurements with cadaveric ligamentous AOD model, a second surgery for combined ligamentous instability of AOD and AAI was performed. This combined model of AOD and AAI could be implemented successfully in all six cadavers confirmed by ADI (Fig 1D). Dural sac was preserved intact in all cases, confirmed by myelographic measurements (Fig 1B, 1C and 1D).

### Compression on dural sac

**Fig 2:**
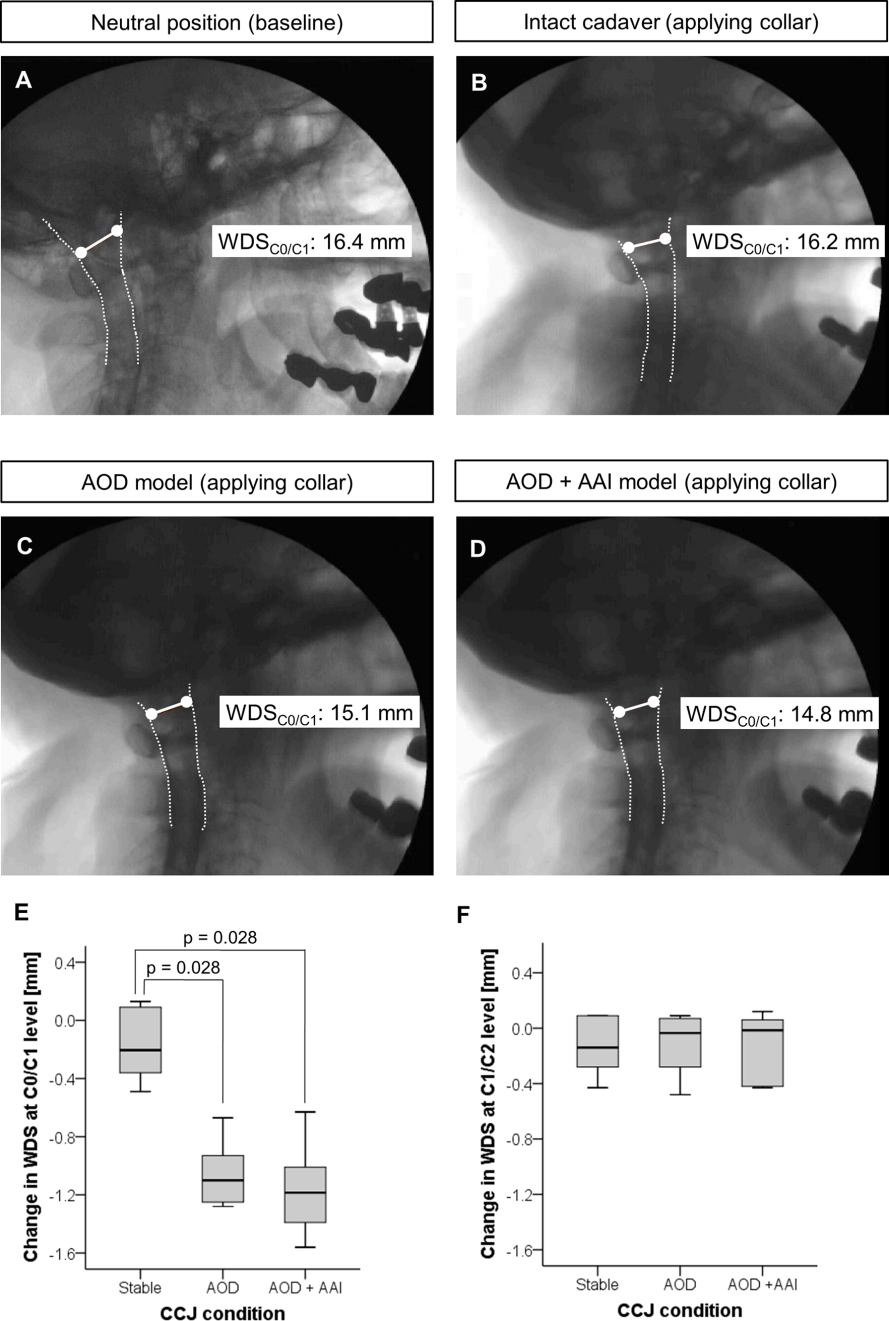
Compression of dural sac measurement. Myelographic views from fluoroscopy data showed WDS in neutral position (A, measured as baseline), and narrowest WDS during cervical collar application on stable CCJ (B), AOD model (C) and combined model of AOD and AAI (D). Overall changes in WDS at C0/C1 level (E) and C1/C2 level (F).

Compared to the stable CCJ condition (Fig 2B), application of cervical collars in the AOD model (Fig 2C) resulted in a significant (p = 0.028) dural sac compression of -1.1 mm (-1.3 to -0.7 mm) at C0/C1 level (Fig 2E). In the combined model of AOD and AAI (Fig 2D) the dural sac was significantly (p = 0.028) compressed of -1.2 mm (-1.6 to -0.6 mm) at C0/C1 level compared to the stable CCJ condition (Fig 2E).

The analysis of change in WDS at C1/C2 level did not show any significant compression on the dural sac in the AOD model as well as in the combined model of AOD and AAI (p = 0.893 and p = 0.833, respectively; Fig 2F).

### Relative intervertebral motion

#### Angulation

**Fig 3:**
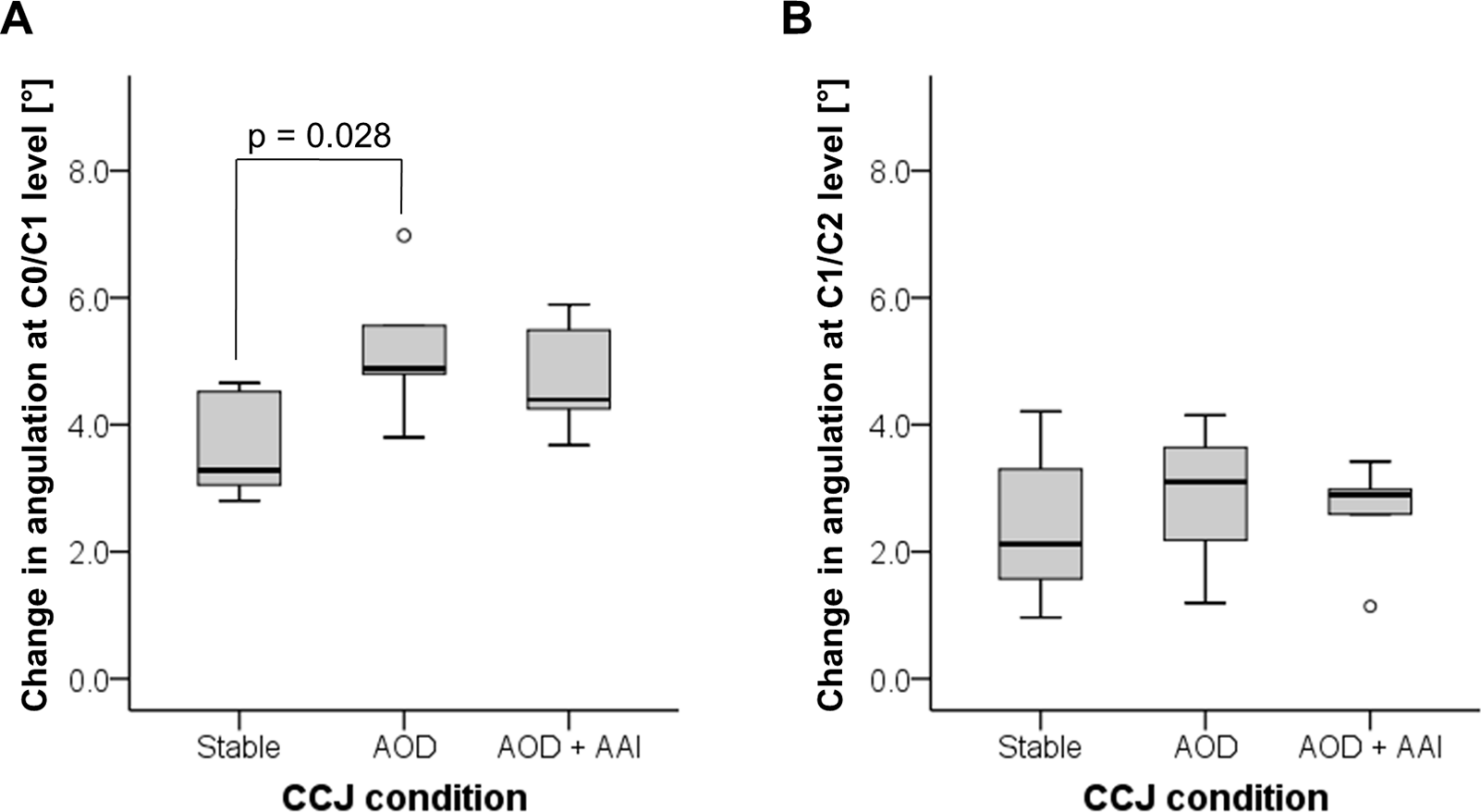
Angulation measurement. The change in angulation from baseline at C0/C1 level (A) and C1/C2 level (B) during cervical collar application.

Compared with stable CCJ, a significant (p = 0.028) increased angulation (Fig 1E; A0) of 4.9° (3.8–7.0°) was measured at C0/C1 segment in the AOD model (Fig 3A). In the more unstable CCJ condition of the combined model with AOD and AAI, there was no significant (p = 0.116) difference in the change of angulation at C0/C1 level (Fig 3A). At C1/C2 level, intervertebral motion by the means of angulation (Fig 1E; A1) was not increased in both of the models (AOD: p = 0.345, AOD + AAI: p = 0.463; Fig 3B).

#### Distraction

Measuring the distraction (Fig 1E; Di) in the upper cervical spine during the application of a cervical collar, C1/C2 level was pulled apart 0.91 mm (0.64 to 1.75 mm) in case of stable CCJ condition. No significant changes have been seen in the AAO model (0.66 mm; p = 0.273) or in the combined model of AOD and AAI (1.10 mm; p = 0.500) as shown in S1 Fig.

### Overall motion of cervical spine

**Fig 4:**
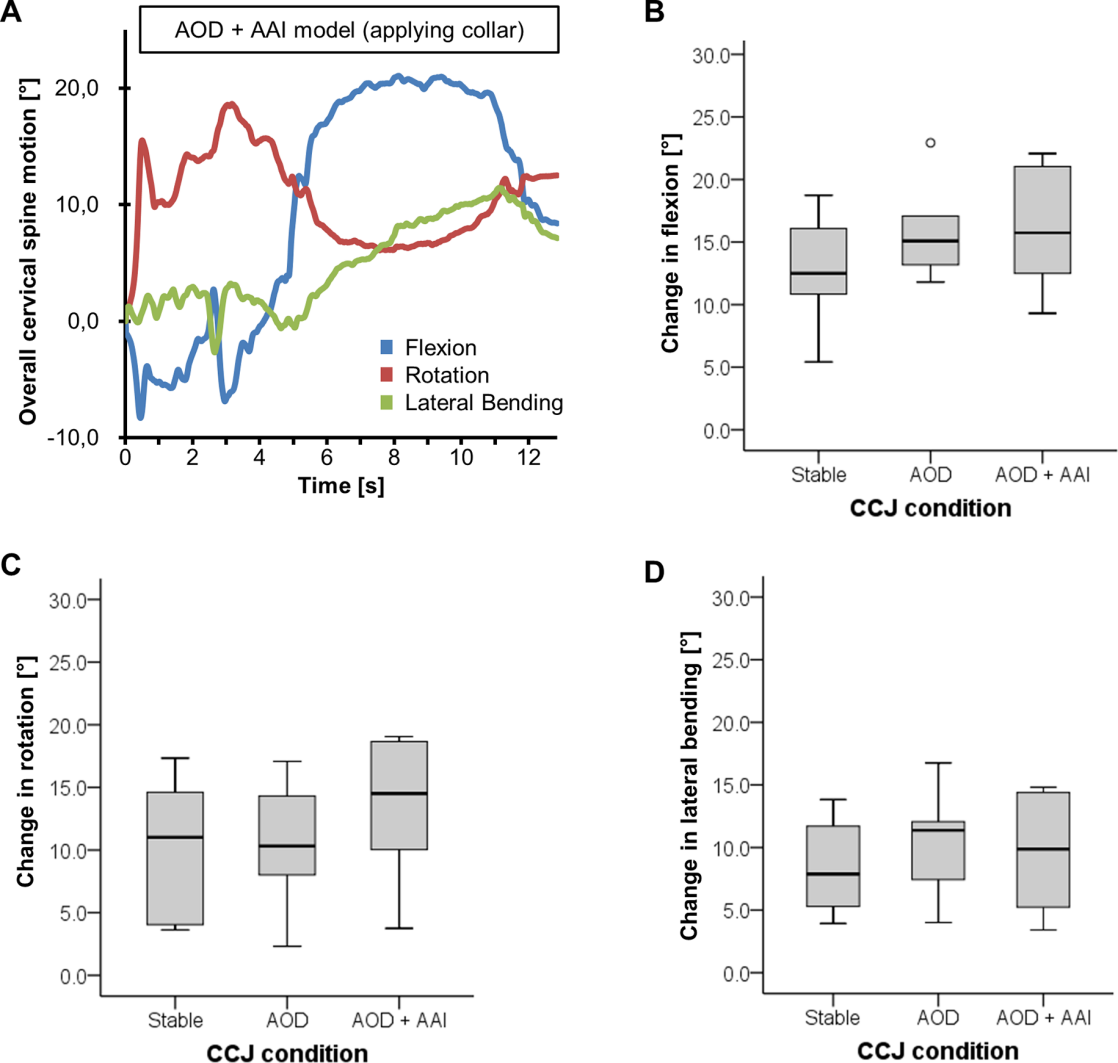
Spinal motion measurement. Overall motion of cervical spine during cervical collar application in 3-dimention orientation (A). Mean values of flexion (B), rotation (C), and lateral bending (D).

Overall cervical spine movement was measured during collar application. Fig 4A shows a representative measurement during the procedure of applying a collar to a cadaver with combined instability at C0/C1 and C1/C2 level (AOD + AAI): The cervical spine was first extended (blue line; negative values) before put in flexion. Furthermore, the cervical spine was rotated (red line) and laterally bended (green line) to the right (positive values). Mean overall cervical spine flexion during collar application was 12.5° (5.4–18.7°) in cadavers with a stable CCJ (Fig 4B). In case of AOD or combined instability AOD and AAI, overall cervical spine flexion did not change significantly to 15.1° (11.8–22.9°; p = 0.249) and 15.7° (9.3–22.1°; p = 0.249), respectively (Fig 4B).

During the procedure of applying a cervical collar rotation of 11.0° (3.6–17.3°) occurs in cadavers with stable CCJ condition (Fig 4C). There was no increase of rotation in the AOD model (10.3°; 2.3–17.1°; p = 0.753) or the combined model of AOD and AAI (14.5°; 3.8–19.1°; p = 0.116).

Measuring of lateral bending reveals movement of 7.9° (3.9–13.8°) if CCJ was stable (Fig 4D). In case of instability, 11.4° (4.0–16.8°; p = 0.345) and 9.9° (3.4–14.8°; p = 0.600) lateral bending motion have been measured in the AOD model and the combine model of AOD and AAI, respectively (Fig 4D).

### Comparison between the two upper segments of cervical spine

**Fig 5:**
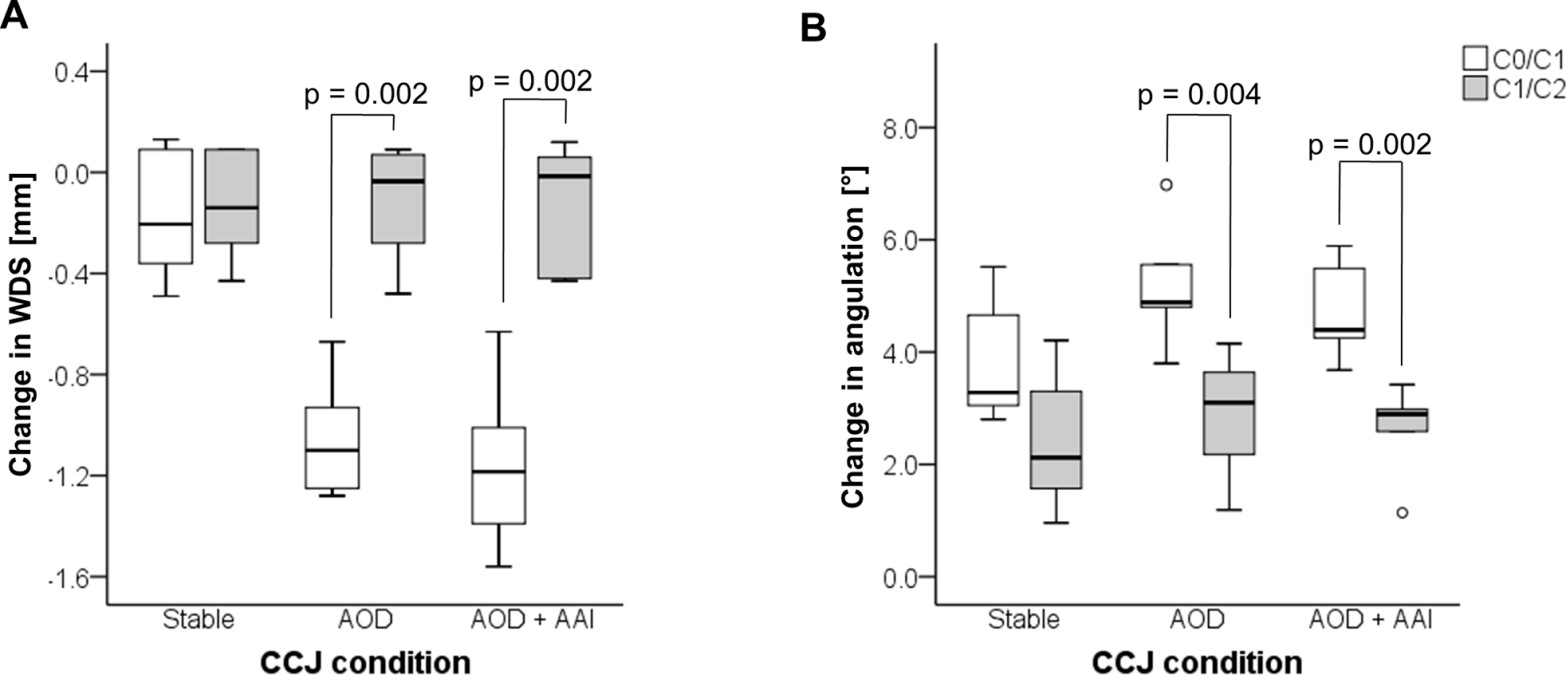
Comparison between C0/C1 segment and C1/C2 segment. Changes in WDS (A) and angulation (B) at different levels of the upper cervical spine during cervical collar application.

Comparisons for change in WDS and angulation between C0/C1 segment and C1/C2 segment in each CCJ condition (Table 1) revealed significantly more compression on the dural sac at the C0/C1 level in both models (AOD: p = 0.002, AOD + AAI: p = 0.002; Fig 5A). Furthermore, intervertebral angulation is significantly increased at C0/C1 level (AOD: p = 0.004, AOD + AAI: p = 0.002; Fig 5B).

**Table 1:** Data of the comparison between the two upper segments of the cervical spine in all CCJ conditions.

	C0/C1 level	C1/C2 level	p-value
**Change of WDS in stable CCJ [mm]**	-0.2 (-0.5 to 0.1)	-0.1 (-0.4 to 0.1)	0.999
**Change of WDS in AOD [mm]**	-1.1 (-1.3 to -0.7)	0.0 (-0.5 to 0.1)	0.002
**Change of WDS in AOD + AAI [mm]**	-1.2 (-1.6 to -0.6)	0.0 (-0.4 to 0.1)	0.002
**Change of angulation in stable CCJ [°]**	3.3 (2.9 to 4.7)	2.1 (1.0 to 4.2)	0.093
**Change of angulation in AOD [°]**	4.9 (3.8 to 7.0)	3.1 (1.2 to 4.1)	0.004
**Change of angulation in AOD + AAI [°]**	4.4 (3.7 to 5.9)	2.9 (1.1 to 3.4)	0.002

See S1 Table for detailed statistical analysis.

## Discussion

Millions of trauma patients are equipped with a cervical collar by emergency medicine personnel every year [22]. Usually, in the prehospital setting the existence of a cervical spine injury reveals unclear. Studies suggest that spinal injuries are the most underestimated injuries in trauma patients [60, 61]. Thus, application of a cervical collar remains nowadays a standard procedure in prehospital trauma care, recommended by recent protocols [20] and guidelines [21].

### Dural sac compression

One of the most severe injuries of the upper cervical spine is AOD. Current studies suggest that AOD may be often combined with an additional AAI [9, 10, 14–17]. However, in any case AOD seems to be responsible for a high mortality [5, 16, 18]. The current study shows that in case of such a severe ligamentous injury of the upper cervical spine the compression of the dural sac is significantly increased during the application of a cervical collar. Compression of the dural sac up to 1.6 mm were measured in the current study. It remains unclear what exact amount of compression of the dural sac will cause spinal cord damage. Eismont et al. postulated that at the C2 level a reduction of the spinal canal’s width of 2 mm may lead to neurological deficits and reduction of 3.4 mm may lead to complete tetraplegia [62]. Since we did not measure the spinal canal’s width but direct compression on the dural sac, spinal cord damage in our trauma models during cervical collar application could not be completely ruled out. Furthermore, additional bony instability such as C1 and C2 fractures that have not been tested in the current study can increase compression on the spinal canal. Especially in patients suffering from degenerative conditions such as rheumatoid arthritis or ankylosing spondylitis spinal canal may be additionally narrowed [63]. However, from clinical experience injuries of the upper cervical spine that are highly suspected to be unstable due to immense dislocation and stenosis of the spinal canal could be associated without any neurological deficits [64]. One reason for this phenomenon could be the mid-sagittal diameter of subarachnoid space within the dural sac at C0/C1 level that has been reported to be 2.5–5.4 mm in anterior aspect and 3.8–6.5 mm in posterior aspect [59]. According to the results of our study, spinal cord damage caused by the application of a cervical collar in patients with injury only at C0/C1 level seems to be unlikely. But taking into account the reported large individual variations in subarachnoid space diameter general conclusions are not allowed.

### Cervical spine motion

As mentioned before, cervical collars are applied to restrict the cervical spine in a neutral position and to protect the cervical spinal cord from further movement during transport. According to our results, maximum flexion of the cervical spine up to 22.9° has been documented during the application of a cervical collar by professional emergency doctors in case of ligamentous upper cervical spine injury. Furthermore, up to 19.1° of rotation and up to 16.8° of lateral bending were measured in our cadaver models during the application of a cervical collar. This overall motion of the cervical spine results in a significant increased angulation at C0/C1 level. Thus, movement of the cervical spine during collar application can add risks to patients with AOD. However, even if the collar is applied, further movement of up to more than 30° is possible as reported by the literature [65]. Since patients with rheumatoid arthritis demonstrated more cord impingement during flexion than during other motions [66], increased angulation could at C0/C1 level should be avoided.

Other cadaver studies found a severe distraction between C1 and C2 during the application of a cervical collar in an upper cervical spine injury model [24]. These data could not be confirmed by our study. The main reason for this discrepancy may be differences in the traumatic cadaver model. Ben-Galim et al. added an odontoid fracture to their model [24] whereas the current study focuses on a ligamentous instability model without any bony fractures.

### Cadaveric CCJ instability models

Recent studies of cadaver biomechanics reported a non-significant difference in cervical spine motion between intact fresh cadavers and living patients in both stable and unstable cervical spine [37–40]. Thus, results of fresh cadaver studies seem to be of some relevance to the clinical situation. However, absence of the protective effect of active muscles and some other biomechanical properties of soft tissues and cervical joints in fresh cadavers will never fully represent living patients.

In the current study we developed two new basic models of ligamentous instability of the upper cervical spine. Both models have been evaluated by anatomical measurements in fluoroscopy images as described by the literature [43–45]. Thus, the current study provides two traumatic cadaver models for further tests on purely ligamentous instability of the upper cervical spine. To our knowledge there is no model of ligamentous AOD nor of combined ligamentous instability of AOD and AAI described so far. Since instability of the C0/C1 and C1/C2 levels seem to be a common combination of injury [9, 10, 14–17], further studies should focus on this combined model. Despite the fact that the spinal canal is wider at the upper level of C0/C1 [59], significant more compression of the dural sac as well as significant increased angulation have been seen in the direct comparison of the two levels of injury.

### Limitations

Caused by the posterior surgical approach alar ligaments could not be severed. Thus, one of the major stabilizer of the C0/C1 level [41, 42] is remaining in the described models limiting the created instability of the upper cervical spine. Furthermore, the current study is limited to some extend based on the study design: Analysis of the fluoroscopy images could not be blinded completely towards the CCJ condition since BDI and ADI values were too obvious. Thus, investigator bias could not be excluded completely.

## Conclusion

The current study determined that applying a cervical collar in general will cause immense three-dimensional movement. In case of instability of the upper cervical spine, this leads to a dural sac compression and thus to possible damage to the spinal cord. Especially in patients with a cervical spine injury and additional degenerative cervical spinal stenosis other possibilities of cervical spine immobilization should be considered.

## Supporting information

S1 Fig Distraction measurement. Changes in C1/C2 distraction during cervical collar application.
(TIF)

S1 Table Results of complete statistics analysis. (XLSX)

S1 Trial Register Details about the study registration in the German Clinical Trials Register. (PDF)
