# Supplementary figures and images for: Motion and dural sac compression in the upper cervical spine during the application of a cervical collar in case of unstable craniocervical junction—A study in two new cadaveric trauma models
Source: PLoS One. 2018 Apr 6;13(4):e0195215. doi: 10.1371/journal.pone.0195215 (PMC5889057; doi:10.1371/journal.pone.0195215)

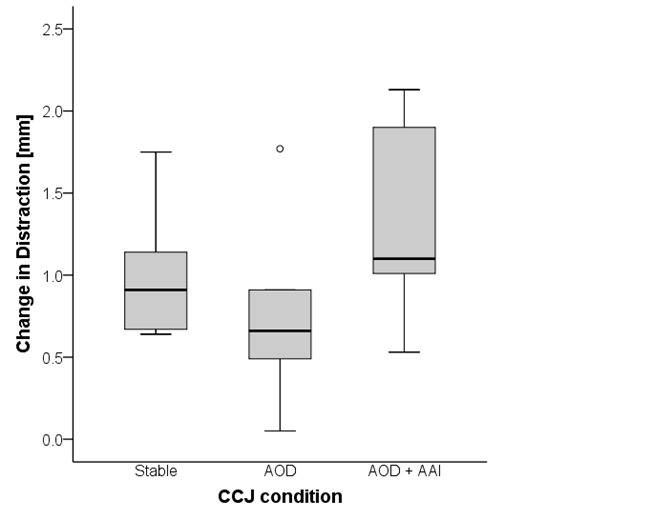

Supplement: S1 Fig — Changes in C1/C2 distraction during cervical collar application. (TIF) [file pone.0195215.s001.tif]
