# Supplementary material for: Motion and dural sac compression in the upper cervical spine during the application of a cervical collar in case of unstable craniocervical junction—A study in two new cadaveric trauma models
Source: PLoS One. 2018 Apr 6;13(4):e0195215. doi: 10.1371/journal.pone.0195215 (PMC5889057; doi:10.1371/journal.pone.0195215)
Supplement: S1 Trial Register — (PDF) [file pone.0195215.s003.pdf]

Deutsches Register Klinischer Studien
German Clinical Trials Register

PLEASE NOTE: *This trial has been registered retrospectively.*

Trial Description

Title

Cadaveric study of movement in the unstable upper cervical spine during emergency management: tracheal intubation and cervical spine immobilization (SPINEMOTION-study)

Trial Acronym

SPINEMOTION-study

URL of the trial

[---]*

Brief Summary in Lay Language

This study will describe the change of cervical spinal canal's space and the simultaneous motion of injured segment during tracheal intubation and cervical collar application. These findings help us to detect the risks and benefits of two kinds of airway management strategies, and provide useful information to improve the immobilization strategy.

Brief Summary in Scientific Language

The study will evaluate two interventions: (i) the change of spinal canal's space during two kinds of airway management strategies (laryngoscopy and intubation, laryngeal tube insertion) in the presence of unstable upper cervical spine injury, and (ii) the change of spinal canal's space during application of a cervical collar in the presence of unstable upper cervical spine injury.Development of cadaveric trauma models
A) Atlanto-occipital dislocation (AOD) cadaveric model
To our knowledge, there is no cadaver study that has formally reported to create an AOD model in an intact cadaver. Thus we attempt to develop an original cadaveric model by referring to the studies about anatomical structure. Previous anatomical studies have documented that the rupture of both tectorial membrane and alar ligament may play a critical role in causing an AOD, and superior band of cruciate ligament also provides structural support for the atlanto-occipital junction. A posterior surgery is performed on a cadaver by severing the tectorial membrane, alar ligament and superior band of cruciate ligament to develop an AOD cadaveric model.
The model will be confirmed during flexion and extension by lateral video fluoroscopy. According to the consensus statement of measurement for upper cervical spine injuries, the value of basion-dental interval (BDI) or basion-posterior axial line interval (BAI) is measured more than 12 mm is highly suggestive of AOD (the exact method of measurement is described below).

B) Type II odontoid fracture cadaveric model
We will make a transverse incision about half inch in the posterior pharynx via

transoral approach, then perform an osteotomy in the base of odontoid to create a type II odontoid fracture under fluoroscopy.
The model is confirmed when a fracture at the odontoid's base is documented during flexion and extension by lateral video fluoroscopy.

Myelography
The cadavers are positioned prone on a table, a mini-incision surgery is performed to expose thecal sac in upper thoracic spine via posterior approach, then make a subarachnoid space puncture and place a tube towards upper cervical spine, the contrast (Omnipaque, 240mg/ml) is pump injected through the tube into thecal sac. This method of puncture ensures the contrast has sufficiently worked in cervical spinal canal, followed by video fluoroscopy.

BDI or BAI
According to the consensus statement of measurement for upper cervical spine injuries, BDI as measured through the distance between basion and dental, and BAI as measured through the distance between basion and the vertical line along the posterior border of C2.
Both BDI and BAI are recommended by Spine Trauma Study Group as standardize measurement techniques to describe the spatial relationship between occiput and atlas, one of these values more than 12 mm is highly suggestive of AOD.

Angulation
The angle of intersection of reference lines on each vertebral body is measured as angulation of each cervical spine motion segment. The position before maneuvers record as neutral position, we define extension as positive value and flexion as negative value.

Distraction of C1-C2.
Distraction is measured as the perpendicular distance between the posterior ring of C1 and the superior spinolaminar line of C2.

Movement of the whole cervical spine
We use wireless human motion tracker (Xsens Technologies, Enschede, Netherlands) to capture the movement of cervical spine during tracheal intubation and cervical collar application. We assess the movement of the whole cervical spine by measuring the change in angulation of the head relative to the trunk. Two inertial measurement units (IMUs) are placed on the forehead and sternum of each cadaver, and the state of cadaver positioned supine on a table before maneuvers is recorded as the neutral state. The change of angulation between the motion state and the neutral state is measured as the movement of the whole cervical spine, and we define extension as positive value and flexion as negative value.

Organizational Data

- DRKS-ID: **DRKS00010499**
- Date of Registration in DRKS: **2016/09/12**
- Date of Registration in Partner Registry or other Primary Registry: **[---]***
- Investigator Sponsored/Initiated Trial (IST/IIT): **yes**
- Ethics Approval/Approval of the Ethics Committee: **Approved**
- (leading) Ethics Committee Nr.: **837.156.16 (10475) , Ethik-Kommission bei der Landesärztekammer Rheinland-Pfalz**

Secondary IDs

Health condition or Problem studied

- Free text: **upper cervical spine injuries**

Interventions/Observational Groups

- Arm 1: **In Cadaver with unstable upper cervical spine injury the change of spinal canal's space measured by using myelography (an X-ray study that involves the injection of a dye into the spinal canal to assess the nerve roots. A needle is injected into the thecal sac containing the nerve roots with radiopaque dye that will highlight the nerve areas in the X-ray image) is beein evaluated during two kinds of airway management strategies (laryngoscopy and intubation, laryngeal tube insertion) and application of a cervical collar**

Characteristics

- Study Type: **Non-interventional**
- Study Type Non-Interventional: **Other**
- Allocation: **Single arm study**
- Blinding: **[---]***
- Who is blinded: **[---]***
- Control: **Uncontrolled/Single arm**
- Purpose: **Prevention**
- Assignment: **Single (group)**
- Phase: **N/A**
- Off-label use (Zulassungsüberschreitende Anwendung eines Arzneimittels): **N/A**

Primary Outcome

Goal of the study is, to evaluate two interventions: (i) the change of spinal canal's space during two kinds of airway management strategies (laryngoscopy and intubation, laryngeal tube insertion) in the presence of unstable upper cervical spine injury, and (ii) the change of spinal canal's space during application of a cervical collar in the presence of unstable upper cervical spine injury.

Secondary Outcome

NA

Countries of recruitment

- DE **Germany**

Locations of Recruitment

- University Medical Center **Heidelberg und BG Klinikum Ludwigshafen**

Recruitment

- Planned/Actual: **Actual**
- (Anticipated or Actual) Date of First Enrollment: **2016/07/29**
- Target Sample Size: **10**
- Monocenter/Multicenter trial: **Multicenter trial**
- National/International: **National**

Inclusion Criteria

- Gender: **Both, male and female**
- Minimum Age: **18 Years**
- Maximum Age: **no maximum age**

Additional Inclusion Criteria

N.A.

Exclusion criteria

N.A.

Addresses

- **Primary Sponsor**

  BG Klinik Ludwigshafen und Uniklinik Heidelberg
  Ludwig Gutmann Str.13

Primary Sponsor

BG Klinik Ludwigshafen und Uniklinik Heidelberg
Ludwig Gutmann Str.13
67701 Ludwigshafen
Germany

Telephone: **[---]***
Fax: **[---]***
E-mail: **[---]***
URL: **[---]***

- **Contact for Scientific Queries**

BG Klinik LudwigshafenKlinik für Unfallchirurgie und OrthopädieUnfallchirurgische Klinik an der Universität HeidelbergLudwig-Guttmann-Str. 1367071 Ludwigshafen
Mr. Dr.Dr. Michael Kreinest
Ludwig-Guttmann-Str. 13
67701 Ludwigshafen
Germany

Telephone: **Telefon: 0621-6810-0**
Fax: **[---]***
E-mail: **michael.kreinest at bgu-ludwigshafen.de**
URL: **[---]***

- **Contact for Public Queries**

BG Klinik LudwigshafenKlinik für Unfallchirurgie und OrthopädieUnfallchirurgische Klinik an der Universität HeidelbergLudwig-Guttmann-Str. 1367071 Ludwigshafen
Mr. Dr.Dr. Michael Kreinest
Ludwig-Guttmann-Str. 13
67701 Ludwigshafen
Germany

Telephone: **Telefon: 0621-6810-0**
Fax: **[---]***
E-mail: **michael.kreinest at bgu-ludwigshafen.de**
URL: **[---]***

Sources of Monetary or Material Support

- **Institutional budget, no external funding (budget of sponsor/PI)**

BG Klinik Ludwigshafen und Uniklinik Heidelberg
Ludwig Gutmann Str.13
67701 Ludwigshafen
Germany

Institutional budget, no external funding (budget of sponsor/PI)

BG Klinik Ludwigshafen und Uniklinik Heidelberg
Ludwig Gutmann Str.13
67701 Ludwigshafen
Germany

Telephone: **[---]***
Fax: **[---]***
E-mail: **[---]***
URL: **[---]***

Status

- Recruitment Status: **Recruiting ongoing**
- Study Closing (LPLV): **[---]***

Trial Publications, Results and other documents

** This entry means the parameter is not applicable or has not been set.*
